# Design of Fear and Anxiety of COVID-19 Assessment Tool in Spanish Adult Population

**DOI:** 10.3390/brainsci11030328

**Published:** 2021-03-05

**Authors:** Juan Gómez-Salgado, Regina Allande-Cussó, Sara Domínguez-Salas, Juan Jesús García-Iglesias, Valle Coronado-Vázquez, Carlos Ruiz-Frutos

**Affiliations:** 1 Department of Sociology, Social Work and Public Health, Faculty of Labour Sciences, University of Huelva, 21007 Huelva, Spain; juanjesus.garcia@dstso.uhu.es (J.J.G.-I.); frutos@uhu.es (C.R.-F.); 2 Safety and Health Postgraduate Programme, Universidad Espíritu Santo, 092301 Guayaquil, Ecuador; 3 Nursing Department, University of Seville, 41009 Seville, Spain; 4 Department of Psychology, Universidad Loyola Andalucía, 41704 Dos Hermanas, Spain; sdominguez@uloyola.es; 5 Illescas Health Centre, Castilla-La Mancha Health Service, 45200 Toledo, Spain; mvcoronado@msn.com; 6 Bioethics Group of the Society of Family and Community Medicine of Madrid (SoMaMFyC), 28004 Madrid, Spain

**Keywords:** anxiety, COVID-19, public health, questionnaire design, Delphi technique, stress, fear

## Abstract

The aim of this study was to develop a specific scale to measure anxiety and fear levels in the general Spanish population. For this, a transcultural adaptation to Spanish of the fear of coronavirus disease 2019 (COVID-19) scale, in its original version of 10 items, was carried out. Then, the Anxiety and Fear of COVID-19 Assessment Scale (AMICO, for its acronym in Spanish) was designed by translating the tool and Delphi technique into three rounds. Ten experts participated voluntarily, and inter-observer match rates and the reliability study of the designed scale were calculated. A pilot study was carried out with the final version of the scale for the validity and reliability study. The instrument did not raise problems in semantic and cultural terms during the first and second rounds of the translation process, with an overall weighted Kappa value of 0.9. In the third round, eight new items were designed and consensual, obtaining a weighted overall value of 0.89. The pilot study sample was made up of 445 subjects, of which 60.3% were women with a mean age of 46.2 years. The final version consisted of 16 items, 2 factors, and a Cronbach’s alpha value of 0.92. The AMICO scale was developed to assess the level of anxiety and fear of COVID-19 and proved to be valid and reliable for its use in the adult Spanish population.

## 1. Introduction

The emergence of the new severe acute respiratory syndrome coronavirus 2 (SARS-CoV-2) coronavirus has been a major challenge for the Spanish public health service and the rest of the world’s powers. We are faced with a disease, coronavirus disease 2019 (COVID-19), of which the scientific community is gradually learning its treatment, transmission capacity, and lethality [1]. The sense of widespread uncertainty caused by this situation, increased by mobility and social contact restrictions imposed in countries such as Spain [2], is adversely affecting different areas of people’s lives and mental health in a particular way [3,4,5,6].

Historical epidemics, such as those caused by the plague and cholera, brought with them scenes of panic and intense fear due to the initial ignorance about their correct approach and their devastating consequences on affected populations [7]. Fear, understood as a cognitive response to a threat [8], favors the adaptation of the human being to certain dangers, but if it remains over time or becomes characteristic of the individual, it can predispose the emergence of physical diseases and/or psychological disorders, or aggravate previous pathologies [9]. In the current case at hand, the data warn of the negative impact of the COVID-19 pandemic on people’s psychological well-being, with increased levels of stress, anxiety, and depression being observed [10,11,12,13]. In addition to the magnitude of the psychological consequences of COVID-19, its prolongation over time has also been noted, coining the term “coronaphobia” to designate those long-term mental illnesses associated with the pandemic, and in which fear and emotional and social tension towards COVID-19 predominate when we look at the harmful reactions and poor coping mechanisms [14].

In order to assess citizens’ fear of COVID-19 and facilitate preventive and further treatment efforts, Ahorsu et al. [15] designed the Fear of COVID-19 scale (FCV-19), which has been validated in the elderly Iranian population. Initially, the instrument consisted of 10 items that, after their psychometric study, eventually resulted in a one-dimensional scale structure composed of seven items and which has adequate psychometric properties [15]. Following its publication, FCV-19 has been validated in different countries, such as Italy [16], the United States [17], Turkey [18], Paraguay [19], Poland [20], or Saudi Arabia [21]. In Spain, Martínez-Lorca et al. [22] have studied its psychometric properties, also determining the existence of a one-dimensional structure and pointing out the reliability of this instrument, albeit in a sample of 606 university students from the Autonomous Community of Castilla la Mancha, maintaining the seven items. In their study, they have obtained evidence in favor of the concurrent validity of the FCV-19 scale by correlating positively with the State–Trait Anxiety Inventory (STAI) [23]. However, the tool was validated in a specific population, university students, which does not represent the characteristics of the adult Spanish population in its entirety.

This research arises from the need for a measurement instrument that allows us to estimate both the levels of fear and anxiety towards COVID-19 in the general adult population in Spain. Therefore, the objective of this work was the development of a scale for this purpose, starting with the design of 10 items carried out by the panel of experts that participated in the study by Ahorsu et al. [15].

## 2. Materials and Methods

### 2.1. Design

The transcultural adaptation into Spanish of the 10 items that were created and designed by the panel of experts that participated in the study by Ahorsu et al. [15] was carried out. With this, the Anxiety and Fear of COVID-19 (AMICO) Assessment Scale was designed and validated during October 2020. Likewise, a descriptive psychometric pilot study was developed for the validity and reliability assessment.

### 2.2. Instrument

The instrument used as the starting point of the study was the 10-item version on which the study by Ahorsu et al. [15] was based and which evaluates the scary construct of COVID-19 with a Likert-type measurement scale of five points. The answers are “1 totally disagree”, “2 disagree”, “3 neutral”, “4 agree”, and “5 totally agree”. The minimum possible score is 10 points, and the maximum score is 50 points [15].

### 2.3. Participants

To achieve the objective of the research, according to the contributions by Epstein et al. [24], two different groups of participants were formed: on the one hand, the panel of experts who agreed to participate in the study consisted of 10 professionals and researchers from different Spanish universities with an academic level of Doctor or Official Master, whose areas of knowledge were public health, family medicine, clinical psychology, nursing, sociology, and social work. Second, for the pilot study, data were collected on a sample of 445 subjects over the age of 18, who were Spanish and residing in Spain. A non-probabilistic snowball sampling process was executed. The sample size required for pilot studies [25], relative to the tool items, was of 180 subjects, although data from 445 subjects were eventually collected.

The sample of experts was accessed through personalized emails that contained all the information and objectives of the study as well as a link to accept and participate.

Regarding the pilot study sample, it was accessed by emails to different groups and disseminated via social networks (WhatsApp©, Facebook©, Twitter©, and LinkedIn©). The email message contained all the information and objectives related to the study, as well as a link to accept participating in the study.

### 2.4. Procedure

**Figure 1:**
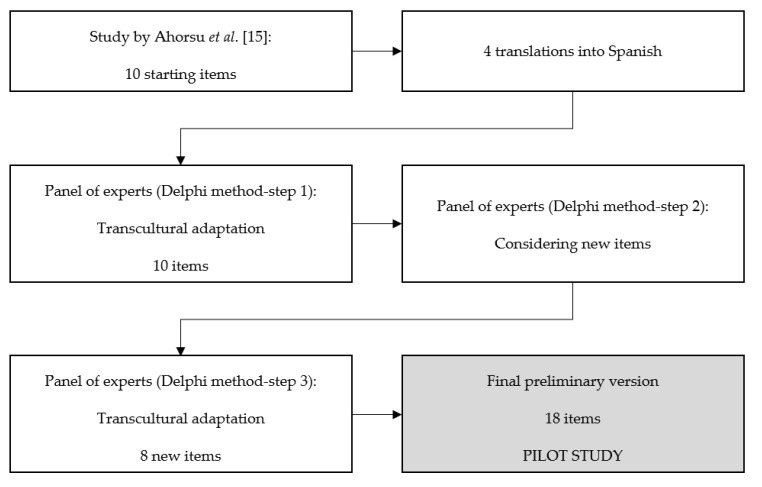
Stages of the questionnaire development process.

Following the proposed classic methodology by Epstein et al. [24], the tool design procedure was developed in four phases (Figure 1):

(a). Translation into Spanish by four translators with a C1 minimum level of English, according to the Common Framework of Reference for Languages [26].

(b). The four Spanish versions were jointly discussed by a panel of 10 experts, using the Google Forms app© (Google, Mountain View, CA, U.S.) and the Delphi technique in three steps:

First step: the experts gave their opinion on the best of the four Spanish translations obtained. In addition, they were asked about the Likert response translation into Spanish and whether they considered this to be the best option for the new response scale. Following the analysis of the collected opinions, consensus was obtained regarding the best translation of the 10 items into Spanish.

Second step: A second round took place to learn the experts’ opinion on the need to include new items in the questionnaire, as it was intended to assess anxiety as well as fear. The opportunity to identify new items related to anxiety medication was also offered, if deemed appropriate, in order to set a scale as comprehensively as possible. Thus, they were freely urged to write down the strictly necessary items, taking into account their area of knowledge and the theoretical and fundamental bases that define the anxiety construct.

Third step: A third round was held with the new items identified in the previous step, and the opinions and final consensus obtained were analyzed.

(c). The preliminary version of the questionnaire, adapted into Spanish and finally made up of 18 items, was submitted to a new assessment by the research team with the aim of studying its applicability and agreeing on the final version to be tested.

(d). A pilot study was conducted for the validity and reliability of the AMICO scale.

### 2.5. Variables

The questionnaires sent to the panel of experts, created through the Google Forms tool©, collected sociodemographic variables (sex, age, academic degree, research activity, teaching activity, and area of knowledge), as well as variables of the instrument to evaluate the corresponding instructions (items). On the other hand, the questionnaire sent to the pilot study sample contained sociodemographic variables (sex, age, and country of residence), the scale variable (18 items from the preliminary version of the AMICO scale), and their free opinion regarding the drafting of the proposed items.

### 2.6. Data Analysis

Statistical analyses were implemented using the Statistical Package for Social Sciences (SPSS) v.26 program [27]. Cohen’s inter-observer or Kappa match rates and Cronbach’s alpha calculation were obtained for the reliability study. In addition, an exploratory factorial analysis was executed for the study of the factorial structure of the scale, and the percentage of variance was explained. To do this, the maximum likelihood extraction method and varimax rotation were selected, eliminating items with loads less than 0.5.

### 2.7. Ethical Aspects

This study is part of the IMPACTCOVID-19 project, which aims to assess the impact of the COVID-19 pandemic on the emotional well-being and psychological adjustment of health professionals and the general population in Spain, which obtained due permission from the Ethics and Research Committee of the Regional Government of Andalusia to be implemented (Ref. PI 036/20). The project was performed according to the guidelines of the Declaration of Helsinki.

All subjects in the sample, as well as the panel of experts, confirmed their voluntary and confidential participation in the study through a specific box, where they had to click on the option “I agree to participate”. Otherwise, the application did not allow access to the questionnaire.

## 3. Results

The following are the main results according to the phases of the study:

(a). In the translation phase of the 10 items identified by the panel of experts of the study by Ahorsu et al. [15] for the adaptation of the Fear of COVID-19 scale (FCV-19 scale) to Spanish, none of the translators reported problems during this process, not in semantic or form terms.

(b). During the first round of the Delphi technique, the panel of experts identified which translation, of the possible four, was best expressed for each of the 10 items. In addition, nine experts identified the need to modify the wording of the items by referencing COVID-19 and not the term “coronavirus”, as was reflected in the original English version. All of them commented that this change should be implemented with the aim of making the items more understandable for the Spanish population.

Regarding the tool’s response options, all experts commented on the need to insert a measurement scale from 1 to 10 points where 1 meant fully disagree and 10 totally agree for each item, instead of the Likert scale of the FCV-19 tool, as they understood that quantifying responses on a scale from 1 to 10 would facilitate the completion of the questionnaire.

With regard to consensus, an overall weighted Kappa index value of 0.9 was obtained.

In the second round, experts freely raised up to 17 new items which measured both anxiety and fear, and these were grouped by the research team into eight categories according to semantic content. For each of them, the research team wrote one item and set up a list of eight new items.

In the last round, the third one, the experts agreed on the adequacy and wording of these eight new items, obtaining a weighted overall value of 0.89.

(c). The final version of the designed questionnaire consisted of 18 items (Table 1), which had been re-assessed by the team of researchers, who agreed on its applicability and proceeded to conduct the pilot study. Items 1 to 10 corresponded to the starting items in the Ahorsu et al. [15] study, and 11 to 18 were the new items identified and agreed upon by the experts.

(d). The final version of the questionnaire was piloted on a sample of 445 subjects, all over the age of 18, who were born and residing in Spain. Of this sample, 60% were women with a mean age of 46.2 years (DE = 12.36). Four hundred and forty-five valid records were obtained, and no subject reported the need to modify the wording of the items (Table 2).

The Kaiser–Meyer–Olkin test obtained a value of 0.94, and the Bartlett sphericity test a statistically significant value (*p* < 0.001). Through exploratory factor analysis (EFA), a dimensional matrix of 16 items was extracted, as well as two factors explaining 64.8% of the variance, with a significance level of < 0.05 (Table 3). Items five and seven were removed from the dimensional structure for having loads lower than 0.5. The reliability study offered a total value of α = 0.92, 0.92 for factor 1, and 0.90 for factor 2. The Spanish and English versions of the AMICO Scale have been attached as supplementary files (see Tables S1 and S2).

## 4. Discussion

The objective of the present study was the development of the AMICO scale to assess fear and anxiety regarding COVID-19. The obtained results have provided good Cohen’s Kappa or inter-observer match rates, as well as a high internal consistency index. It has a final structure of 16 items and 2 factors, extracted by an exploratory factor analysis, and which explain 64.8% of the variance.

The design of the scale was based on the 10 items created by the panel of experts that participated in the study by Ahorsu et al. [15]. This list of items, which is more thorough and designed for the general adult population, also included items related to the concern felt towards COVID-19 and to the perception of the prognosis of the disease in public health. Given the claim of designing a reliable scale to be used in the Spanish population, it was decided to use this initial ratio of 10 items as a starting point for the design of the AMICO scale, and not the final version of seven items of the FCV-19 [15] scale. The design was also not based on the version validated in Spanish by Martínez et al. [22] since the study was carried out on samples of university students from the Autonomous Community of Castilla la Mancha in Spain. In doing so, the research team considered that this sample did not represent the characteristics of the adult Spanish population as a whole.

During the second phase of design of the AMICO scale, eight more items were added by consensus of the participating experts, covering issues related to fear of contagion, the presence of negative thoughts when listening to or reading news related to the COVID-19 disease, and even anxiety caused by contradictory information. This addition of new items responded to the willingness of the research team to thoroughly evaluate the behaviors, attitudes, and emotions related to the new disease in the Spanish population. Thus, the aim of the study was to create a scale that measured both anxiety and fear of COVID-19, considering as a starting point the initial 10 items of the study by Ahorsu et al. [15] and to complete them with eight new items in order to improve the completeness of the AMICO scale. Besides, the FCV-19 tool was validated in an Asian population, whose cultural characteristics differ from the Latin population in terms of proximity to other people or contact, so this fact motivated us to consider including those new aspects and items in the AMICO [28] tool.

Regarding the relevance of the design of the AMICO scale, it is worth mentioning the existence of recently published works that demonstrate the relationship between high levels of concern, depression, and anxiety in relation to COVID-19 and the presence of psychological distress in the Spanish population, also being predictors of this morbidity [10,29,30]. In addition, other authors have concluded that higher levels of anxiety and fear relative to COVID-19 are associated with more appropriate and consistent protective behaviors [30,31], although other studies have obtained opposing data [32,33]. In this context, a reliable tool is necessary to evaluate the levels of anxiety and fear of the Spanish adult population in order to design specific psychosocial and multidisciplinary intervention programs. These could reduce the emotional impact of the new disease, improving coping strategies and adherence to preventive behaviors, as well as increasing public health levels. It should be discussed that the Spanish population could score high on the AMICO scale during the acute pandemic situation considering their situation as a “state”, i.e., a response to a specific acute situation, and not as a “trait”, that is, a permanent attribute. Thus, after the implementation of specific intervention program, the obtained scores may decrease, understanding that their origin lies in an acute response to the pandemic situation they are experiencing, not identifying the obtained outcomes as a permanent attribute of the Spanish population. Thus, after the implementation of specific intervention program, the obtained scores will decrease, indicating that the obtained level of anxiety and fear is not related to “trait-related” situations and is, therefore, susceptible to intervention. The AMICO scale could therefore be used as a process and result indicator of such specific intervention programs in relation to the emotional impact of the pandemic in Spain.

This study is a first step in the process of constructing the AMICO scale. A new field study is required for the execution of a confirmatory analysis of the dimensional structure of the scale. In addition, it would also be appropriate to obtain evidence of its convergent and discriminatory validity and to develop a correction scale. Despite having certain limitations, such as the selection of participants through non-probabilistic sampling, this research provides the design of a scale to evaluate the presence of fear and anxiety regarding COVID-19, which is valid and reliable to be applied in the Spanish population. However, further research is needed in order to confirm the dimensional structure. The research group’s claim was to design a reliable tool to be implanted briefly in Spain to identify people and groups susceptible to specific interventions towards improving their health status from a bio-psycho-social perspective. At the same time, these new findings will provide more evidence about the AMICO Scale dimensions, construct validity, and discriminant validity.

## 5. Conclusions

The AMICO scale has proven to be reliable and valid to assess the level of anxiety and fear of COVID-19 in the Spanish population. The instrument developed has a 2-factor dimensional structure, 16 items, with a Likert response of ten points. Therefore, the AMICO scale could be used as a specific assessment tool for fear of COVID-19 in Spain.

## Figures and Tables

**Table 1:** Mean Kappa coefficient by the designed questionnaire items. COVID-19; coronavirus disease 2019.

Items	Mean Kappa Index *
1. I am very afraid of COVID-19	0.89
2. I feel uneasiness when thinking about COVID-19	0.91
3. I am very concerned about getting COVID-19	0.90
4. The COVID-19 disease may cause death, and this worries me	0.86
5. COVID-19 is unpredictable	0.93
6. My hands sweat when I think about COVID-19	0.94
7.I am afraid of dying due to COVID-19	0.92
8. I feel nervous or anxious when watching news or stories about COVID-19 in social networks and other media	0.95
9. I can’t sleep because I am worried about getting COVID-19	0.89
10. My pulse races when I think about getting COVID-19	0.91
11. Contradictory information about coronavirus in social networks and the media makes me feel anxious	0.90
12. I have negative ideas when I hear or read any news related to the disease	0.84
13. I am afraid any relative or friend may get COVID-19	0.92
14. I am worried about how long the pandemic will last	0.87
15. When someone coughs near me, or I consider he or she is too close to me, I am afraid of getting infected	0.84
16. I am worried about being close to or assisting a person that has or may have COVID-19	0.89
17. I feel sad or downcast when I think about the disease and the possibility of getting infected	0.89
18. I feel anxious about going out, or considering going out, to fulfill my daily responsibilities (work, family, etc.)	0.90

* 95% CI and *p* < 0.001.

**Table 2:** Sample profile.

Sex	Female	*n* = 268 (60%)	Total sample *n* = 445
	Male	*n* = 177 (40%)	
Country of residence	Spain	*n* = 445 (100%)	
Age	Mean	46.2	
	Standard deviation	12.36	

**Table 3:** Exploratory factor analysis: rotated factor matrix.

Scale Items	Factors	
	1	2
Item 1		0.742
Item 2		0.659
Item 3		0.784
Item 4		0.790
Item 6	0.768	
Item 8	0.687	
Item 9	0.867	
Item 10	0.857	
Item 11	0.687	
Item 12	0.727	
Item 13		0.710
Item 14		0.634
Item 15		0.683
Item 16		0.640
Item 17	0.753	
Item 18	0.726	

## Data Availability

All data generated in this study are showed in this article, its tables, and figures.

## Supplementary Materials

The following are available online at https://www.mdpi.com/2076-3425/11/3/328/s1. Table S1: AMICO Scale (Spanish version); Table S2: AMICO Scale (English version).

## References

[1] Aljabali A.A.A., Bakshi H.A., Satija S., Metha M., Prasher P., Ennab R.M., Chellappan D.K., Gupta G., Negi P., Goyal R. (2020). COVID-19: Underpinning Research for Detection, Therapeutics, and Vaccines Development. Pharm. Nanotechnol..

[2] Real Decreto 463/2020. Boletín Oficial del Estado, n° 67, 14 de marzo de 2020; p. 25390–25400. https://www.boe.es/eli/es/rd/2020/03/14/463.

[3] Li S., Wang Y., Xue J., Zhao N., Zhu T. (2020). The Impact of COVID-19 Epidemic Declaration on Psychological Consequences: A Study on Active Weibo Users. Int. J. Environ. Res. Public Health.

[4] Cabello F., Sánchez F., Farré J.M., Montejo A.L. (2020). Consensus on Recommendations for Safe Sexual Activity during the COVID-19 Coronavirus Pandemic. J. Clin. Med..

[5] Yuksel B., Ozgor F. (2020). Effect of the COVID-19 pandemic on female sexual behavior. Int. J. Gynecol. Obstet..

[6] Qiu J., Shen B., Zhao M., Wang Z., Xie B., Xu Y.F. (2020). A nationwide survey of psychological distress among Chinese people in the COVID-19 epidemic: Implications and policy recommendations. Sustainability.

[7] Fernández I., Martín C., Páez D., Apalategui J. (1995). Emociones y conductas colectivas en catástrofes: Ansiedad y rumor y conductas de pánico. La Anticipación de la Sociedad. Psicología Social de los Movimientos Sociales.

[8] Beck A., Emery G. (2005). Anxiety Disorders and Phobias: A Cognitive Perspective.

[9] Sylvers P., Lilienfeld S.O., LaPrairie J.L. (2011). Differences between trait fear and trait anxiety: Implications for psychopathology. Clin. Psychol. Rev..

[10] Dominguez-Salas S., Gomez-Salgado J., Andres-Villas M., Diaz-Milanes D., Romero-Martin M., Ruiz-Frutos C. (2020). Psycho-Emotional Approach to the Psychological Distress Related to the COVID-19 Pandemic in Spain: A Cross-Sectional Observational Study. Healthcare.

[11] Gómez-Salgado J., Andrés-Villas M., Domínguez-Salas S., Díaz-Milanés D., Ruiz-Frutos C. (2020). Related Health Factors of Psychological Distress During the COVID-19 Pandemic in Spain. Int. J. Environ. Res. Public Health.

[12] Gómez-Salgado J., Domínguez-Salas S., Romero-Martín M., Ortega-Moreno M., García-Iglesias J.J., Ruiz-Frutos C. (2020). Sense of Coherence and Psychological Distress among Healthcare Workers during the COVID-19 Pandemic in Spain. Sustainability.

[13] Rodríguez-Domínguez C., Carrascal-Caputto B., Durán M. (2020). Anxiety and Intimate Relationships in Times of Lockdown due to COVID-19. Psychol. Trauma.

[14] Watson J. (2020). COVID-19’s Psychological Impact Gets a Name.

[15] Ahorsu D.K., Lin C.Y., Imani V., Saffari M., Griffiths M.D., Pakpour A.H. (2020). The Fear of COVID-19 Scale: Development and Initial Validation. Int. J. Ment. Health Addict..

[16] Soraci P., Ferrari A., Abbiati F.A., Del Fante E., De Pace R., Urso A., Griffiths M.D. (2020). Validation and Psychometric Evaluation of the Italian Version of the Fear of COVID-19 Scale. Int. J. Ment. Health Addict..

[17] Perz C.A., Lang B.A., Harrington R. (2020). Validation of the Fear of COVID-19 Scale in a US College Sample. Int. J. Ment. Health Addict..

[18] Satici B., Gocet-Tekin E., Deniz M.E., Satici S.A. (2020). Adaptation of the Fear of COVID-19 Scale: Its Association with Psychological Distress and Life Satisfaction in Turkey. Int. J. Ment. Health Addict..

[19] Barrios I., Ríos-González C., O’Higgins M., González I., García O., Ruiz N., Castaldelli-Maia J.M., Ventriglio A., Torales J. (2020). Psychometric properties of the Spanish version of the Fear of COVID-19 Scale (FCV-19S). Res. Square.

[20] Reznik A., Gritsenko V., Konstantinov V., Khamenka N., Isralowitz R. (2020). COVID-19 Fear in Eastern Europe: Validation of the Fear of COVID-19 Scale. Int. J. Ment. Health Addict..

[21] Alyami M., Henning M., Krägeloh C.U., Alyami H. (2020). Psychometric Evaluation of the Arabic Version of the Fear of COVID-19 Scale. Int. J. Ment. Health Addict..

[22] Martínez-Lorca M., Martínez-Lorca A., Criado-Álvarez J.J., Armesilla M.D.C., Latorre J.M. (2020). The fear of COVID-19 scale: Validation in spanish university students. Psychiatry Res..

[23] Spielberger C.D., Gorsuch R.L., Lushene R.E., Buela-Casal G., Guillén-Riquelme A., Seisdedos Cubero N. (2011). STAI: Cuestionario de Ansiedad Estado-Rasgo: Manual.

[24] Epstein J., Miyuki R., Guillemin F. (2015). A review of guidelines for cross-cultural adaptation of questionnaires could not bring out a consensus. J. Clin. Epidemiol..

[25] Hertzog M. (2008). Considerations in determining sample size for pilot studies. Res. Nurs. Health.

[26] Ministerio de Educación Cultura y Deporte (2002). Marco Común Europeo de Referencia para las Lenguas: Aprendizaje, Enseñanza y Evaluación.

[27] IBM Corp (2019). IBM SPSS Statistics for Windows, Version 26.0.

[28] Wang R., Hempton B., Dugan J.P., Komives S.R. (2008). Cultural Differences: Why Do Asians Avoid Extreme Responses?. Surv. Pract..

[29] Rodríguez R., Garrido H., Collado S. (2020). Psychological Impact and Associated Factors During the Initial Stage of the Coronavirus (COVID-19) Pandemic Among the General Population in Spain. Front. Psychol..

[30] Tee M., Tee C., Anlacan J., Aligam K., Reyes P., Hurichittham V. (2020). Psychological impact of COVID-19 pandemic in the Philippines. J. Affec. Disord..

[31] Rubin G., Wessely S. (2020). The psychological effects of quarantining a city. BMJ.

[32] Harper C.A., Satchell L.P., Fido D., Latzman R.D. (2020). Functional Fear Predicts Public Health Compliance in the COVID-19 Pandemic. Int. J. Ment. Health Addict..

[33] Wang C., Pan R., Wan X., Tan Y., Xu L., Ho C.S., Ho R.C. (2020). Immediate Psychological Responses and Associated Factors during the Initial Stage of the 2019 Coronavirus Disease (COVID-19) Epidemic among the General Population in China. Int. J. Environ. Res. Public Health.

