# Supplementary material for: Design of Fear and Anxiety of COVID-19 Assessment Tool in Spanish Adult Population
Source: Brain Sci. 2021 Mar 5;11(3):328. doi: 10.3390/brainsci11030328 (PMC8001709; doi:10.3390/brainsci11030328)
Supplement: Supplementary file 1 [file brainsci-11-00328-s001.pdf]

Table S1. AMICO Scale (Spanish version)

Escala AMICO
1. Tengo mucho miedo a la enfermedad COVID-19
2. Me causa incomodidad pensar en la enfermedad COVID-19
3. Me preocupa mucho contraer la enfermedad COVID-19
4. La enfermedad COVID-19 puede ser causa de muerte, y esto me preocupa
5. Me sudan las manos cuando pienso en la enfermedad COVID-19
6. Siento nervios o ansiedad al ver noticias e historias sobre la enfermedad COVID-19 en las redes sociales y medios de comunicación.
7. No puedo dormir porque me preocupa contraer la enfermedad COVID-19
8. Se me acelera el pulso cuando pienso en contraer la enfermedad COVID-19
9. Las contradicciones informativas sobre el coronavirus en medios de comunicación y redes sociales me producen ansiedad
10. Me asaltan pensamientos negativos cuando escucho o leo una noticia relacionada con la enfermedad
11. Me preocupa que algún familiar o amistad pueda contraer la enfermedad COVID-19
12. Me preocupa cuánto va a durar la pandemia
13. Cuando alguien tose cerca de mi o considero que está muy cerca de mi tengo miedo a que me contagie
14. Me da miedo estar cerca o atender a una persona que tiene o puede tener COVID-19
15. Me siento triste o sin fuerzas cuando pienso en la enfermedad y en la posibilidad de contagiarme
16. Me produce ansiedad salir de casa, o pensar en ello, para cumplir con mis obligaciones del día a día (laborales, familiares, etc.)

Table S2. AMICO Scale (English version).

AMICO Scale Items
1. I am very afraid of COVID-19
2. I feel uneasiness when thinking about COVID-19
3. I am very concerned about getting COVID-19
4. The COVID-19 disease may cause death, and this worries me
5. My hands sweat when I think about COVID-19
6. I feel nervous or anxious when watching news or stories about COVID-19 in social networks and other media
7. I can´t sleep because I am worried about getting COVID-19
8. My pulse races when I think about getting COVID-19
9. Contradictory information about coronavirus in social networks and the media makes me feel anxious
10. I have negative ideas when I hear or read any news related to the disease
11. I am afraid any relative or friend may get COVID-19
12. I am worried about how long the pandemic will last
13. When someone coughs near me, or I consider he or she is too close to me, I am afraid of getting infected
14. I am worried about being close to or assisting a person that has or may have COVID-19
15. I feel sad or downcast when I think about the disease and the possibility of getting infected
16. I feel anxious about going out, or considering going out, to fulfill my daily responsibilities (work, family, etc.)
